# Tracking the urban spread of Usutu virus in southern France: Detection across biological and environmental matrices

**DOI:** 10.1371/journal.pntd.0013506

**Published:** 2025-09-02

**Authors:** Rachel Beaubaton, Justine Revel, Laetitia Pigeyre, Karine Bollore, Alexandre Lepeule, Julien Mocq, Christophe de Franceschi, Julien Pradel, Yvon Perrin, David Gomis, Marie Ducousso, Laurie Virolle, Baptiste Chenet, Guillaume Castel, Anne Charmantier, Nathalie Charbonnel, Guillaume Lacour, Olivier Courot, Antoine Mignotte, Yannick Simonin

**Affiliations:** 1 Pathogenesis and Control of Chronic and Emerging Infections (PCCEI), Université de Montpellier, Inserm, Montpellier, France; 2 Ingénierie et Analyse en Génétique Environnementale, Montpellier, France; 3 Altopictus, Pérols, France; 4 CEFE, Univ Montpellier, CNRS, EPHE, IRD, Montpellier, France; 5 CBGP, INRAE, CIRAD, Institut Agro, IRD Université de Montpellier, Montpellier, France; 6 Montpellier Méditerranée Métropole, Montpellier, France; 7 Parc de Lunaret - Zoo de Montpellier, Montpellier, France; The University of the West Indies, JAMAICA

## Abstract

The Usutu virus, a neurotropic *Orthoflavivirus* transmitted by mosquitoes, was first identified in South Africa in 1959 and has progressively spread across Europe over the past two decades. This virus follows an enzootic cycle between mosquitoes and birds, leading to periodic outbreaks that have caused significant bird mortality. Although primarily an avian pathogen, Usutu virus can occasionally infect humans and other mammals who act as incidental or dead-end hosts. The repeated avian epizootics observed in Europe in the last two decades raise concerns about potential zoonotic risks, even though human infections remain rare. In most cases, human infection is either asymptomatic or results in mild symptoms. However, in some instances, Usutu virus has been linked to severe neurological conditions, including encephalitis and meningoencephalitis. The Occitanie region in the south of France is particularly vulnerable to this threat due to its ecosystem, which harbors both competent mosquito vectors and numerous avian hosts that act as amplifying hosts for the virus. We investigated the urban circulation of Usutu virus in the city of Montpellier, where the first human case of infection by this virus in France was previously identified. To assess the presence of Usutu virus, we conducted a repeated cross-sectional study using serological (ELISA, microneutralization) and molecular (RT-qPCR) analyses of captive avifauna, including a longitudinal study of captive birds at the Montpellier zoological park between 2016 and 2024. Additionally, in 2024, we completed our study with avian cloacal swabs, pigeon droppings, rat blood, mosquito faeces, and environmental water samples (dPCR). Our findings revealed active circulation of the Usutu virus in the urban environment over multiple years. Furthermore, we demonstrated the feasibility of detecting the virus in droppings and environmental waters, highlighting the potential of environmental surveillance as a non-invasive and large-scale method. This study contributes to a better understanding of Usutu virus circulation and highlights its established presence in urban areas.

## Author summary

The Usutu virus is transmitted among bird populations by the *Culex pipiens* mosquito and is associated with avian mortality. It can also cause neurological disorders in humans, with cases primarily reported in Europe. Usutu virus poses a threat to both human and bird health, as infections are often asymptomatic and thus difficult to detect, especially given the limited surveillance networks in place. This study, conducted in Montpellier, southern France, aims to establish surveillance networks to assess the distribution of the virus. The region, home to large bird and mosquito populations, is particularly exposed to Usutu virus, with both avian and human cases reported. We have documented its urban circulation in birds, mosquitoes, and water, emphasizing the need for comprehensive monitoring to track its spread and establish an early warning system.

## Introduction

The Usutu virus (USUV) is an arbovirus of the genus *Orthoflavivirus*, from the *Flaviviridae* family. It was first identified and isolated in 1959 in South Africa near the Usutu River, in the mosquito *Culex neavei* (*Diptera*: *Culicidae*). It primarily circulates in Africa and Europe, where it has become endemic in certain areas, such as Germany and Italy [1–4]. USUV primarily infects birds, with species such as house sparrows (*Passer domesticus*) serving as key amplifying hosts [5]. Transmission occurs via the bite of infected mosquitoes, predominantly *Culex pipiens* (the Northern house mosquito) [1]. While many bird species develop subclinical infections, some - particularly blackbirds (*Turdus merula*) and certain captive owls - are highly susceptible and may experience significant mortality during outbreaks [6]. USUV is very similar to the West Nile virus (WNV), another *Orthoflavivirus* belonging to the Japanese Encephalitis Virus serocomplex. Both viruses share similar biological traits, transmission cycle and epidemiology [7]. These viruses are increasingly attracting the interest of the scientific community in recent years due to their growing impact on animal health, but also on human health because of their zoonotic potential. First confirmed USUV cases emerged in 1996 in Italy, and caused a first outbreak among birds, mainly blackbirds (*Turdus merula*) and great grey owls (*Strix nebulosa*), in Vienna, Austria in 2001 [8,9]. The circulation of USUV in Europe has increased considerably over the past two decades, with a major USUV outbreak in 2018 affecting several European countries and causing high mortality in several bird species especially blackbirds (*Turdus merula*), magpies (*Pica pica*) and *Strigiformes*, such as the great grey owl (*Strix nebulosa*) [1,4,10]. Since then, the virus has been linked to widespread infections and high mortality rates among birds across Europe primarily in Hungary, Czechia, Austria, Italy, Switzerland, Belgium, Netherlands, Germany, United Kingdom, and France [11–20]. Originally limited to Southern Europe, USUV distribution area appears to expand northward. Its endemization in Europe likely results from the migratory behaviours of certain bird species, with some species such as the common kestrel (*Falco tinnunculus*) or the lesser whitethroat (*Sylvia curruca*), suspected of introducing the virus in new areas [1,3]. Resident avian species may subsequently contribute to the maintenance and spread of the virus. This is the case for tits (*Paridae*), a group of resident passerine species found in the south of France, as well as pigeons (*Columbidae*), which have been identified in the literature as an urban species susceptible to USUV [21–25]. Infection by USUV in birds can cause severe symptoms, including necrotic and neurological lesions, leading to significant mortality, particularly in certain species such as the common blackbird (*Turdus merula*), great grey owls (*Strix nebulosa*), and house sparrows (*Passer domesticus*) [1,6,9,26]. However, alongside these infections in birds, the vector mosquito *Culex pipiens* sometimes transmits USUV to several mammal species, which are dead-end hosts, including horses, dogs, wild boar, various wild ruminants such as deer and sheep, bats, and humans [1]. Moreover, rodents have been suggested as potential hosts for USUV infections and possible secondary reservoirs of the virus [27]. USUV being primarily an avian virus, it remains unclear whether these different species could play a role in viral transmission. Humans are a known epidemiological dead end, probably because they do not develop sufficient viremia to sustain viral transmission [1,3]. Human disease associated with USUV is typically asymptomatic. However, symptomatic cases may exhibit mild symptoms such as fever, headache, skin rashes, and muscle or joint pain. In a small proportion of cases, neurological complications like encephalitis, meningitis, and meningoencephalitis can appear. To date, approximately thirty cases of neurological complications of varying severity have been reported in Europe [1,3]. The introduction of USUV into Europe follows major migratory routes [3,28]. The first recorded entry from Africa to Spain aligns with the East Atlantic migratory pathway, while its introduction to Central Europe follows the Black Sea/Mediterranean route [3]. As a result, USUV has been classified into eight distinct lineages, categorized as either African (Africa 1/2/3) or European (Europe 1/2/3/4/5) [1].

USUV infections have previously been reported in Southern France, around Montpellier city, in mosquitoes [29,30], birds [31], dogs, horses [30] and in several species of the Montpellier zoological park [20]. Notably, USUV has been detected in several greater rheas (*Rhea americana*), highlighting their particular susceptibility to USUV infection. Human USUV infection in Montpellier was first reported in 2016 in a hospitalized patient with idiopathic facial paralysis [32] and 3% of blood donors were detected seropositive in the same area [30]. All these elements suggest an active circulation of USUV around the city of Montpellier. The Camargue Regional Nature Park and the nearby marshland systems can create highly favourable conditions for the maintenance and circulation of this arbovirus. These semi-aquatic ecosystems along the Mediterranean Sea are characterized by high bird densities, their location along key migration corridors, and a strong presence of vector mosquitoes [30]. Consequently, Montpellier represents a key location for USUV circulation. Monitoring viral circulation is challenging, especially for viruses like USUV with complex transmission cycles. Therefore, to better understand the urban circulation of USUV, we conducted a long-term in-depth study using innovative approaches. We aimed to assess whether the circulation of USUV was continuous over time or characterized by peaks in certain years with periods of undetected circulation in others. We performed a 7-year monitoring of the seroprevalence of captive bird species at the Montpellier zoological park to assess the dynamics of virus circulation. Furthermore, in 2024, we expanded our research to additional urban sites, animal species and methods, such as monitoring cloacal samples in tits, monitoring rat blood, sampling mosquito faeces and pigeon droppings. We also established environmental water monitoring, assuming that virus excretion by birds could allow for environmental detection. Our results show active circulation of the Usutu virus over the 7 years of the study. Additionally, we were able to detect the virus in cloacal swabs, and for the first time in droppings, and in stagnant environmental water. These data suggest that studying virus excretion, especially in environmental water, could be an effective monitoring method for this virus, using less costly and less invasive techniques than those currently employed.

## Methods

### Ethical statement

Avian zoo samples were collected from serum banks or from animals undergoing health check-ups, health programs, or surgical procedures. Capture and handling of rats have been conducted according to the French and European regulations on care and protection of laboratory animals (French Law 2001–486 issued on June 6, 2001 and Directive 2010/63/EU issued on September 22, 2010; Agreement from the C2EA-LR). The CBGP laboratory has approval (F-34-169-001) from the Departmental Direction of Population Protection (DDPP, Hérault, France), for the sampling of rodents and the storage and use of their tissues. For the studies on tits by CEFE: approval number: F 34 172 11, issued in February 2024. No animals were sampled exclusively for the purpose of this study.

### 1. Sampling

The exotic avifauna samples were collected at the Montpellier zoological park, located north of Montpellier (Southern France), in a peri-urban area. The park spans 80 hectares and houses 40 animal species including both birds and mammals from every continent. The analyses were also extended to other locations with no known history of USUV circulation. We therefore tested several environmental water sampling locations around Montpellier for Usutu virus RNA. We collected samples from the Montpellier Ecolotheque, a 4-hectare educational farm located in Saint-Jean-de-Védas (South of Montpellier), which includes traditional farmyard animals and various bird species. We also sampled tits, species known to be susceptible to USUV infection, in research aviaries at the Center for Functional and Evolutionary Ecology (CEFE), located 1 km from the Montpellier zoological park. In collaboration with Montpellier Méditerranée Métropole (3M), we also conducted a targeted analysis of a pigeon loft located on Charles de Gaulle Esplanade, in the heart of the city of Montpellier, examining pigeon droppings following a series of unexplained deaths. Finally, we studied two other sites of mosquito control operation within the Montpellier metropolitan area, to explore the possibility of detecting the presence of the virus in water at locations with a lower concentration of birds compared to the zoo (Fig 4).

**Fig 1:**
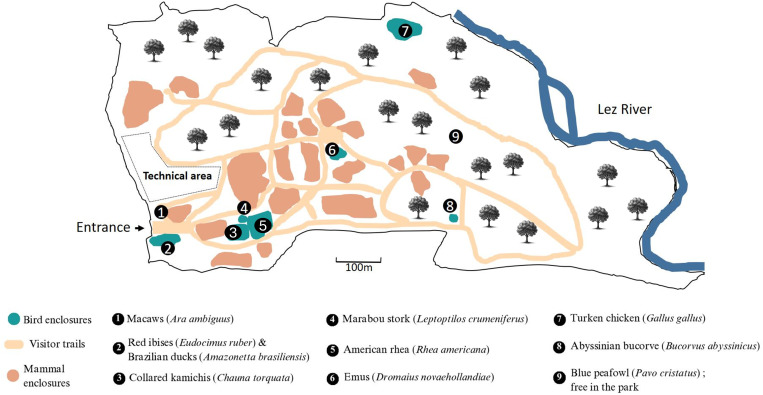
Map of the bird enclosures within the Lunaret zoological park, northern Montpellier. Figure created using canva.com.

**Fig 2:**
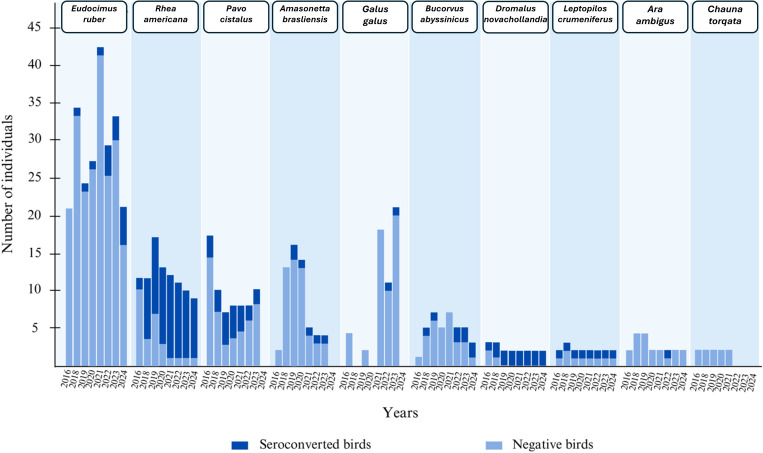
Orthoflavivirus ELISA positive zoo birds by species between 2016 and 2024. Figure created using canva.com.

**Fig 3:**
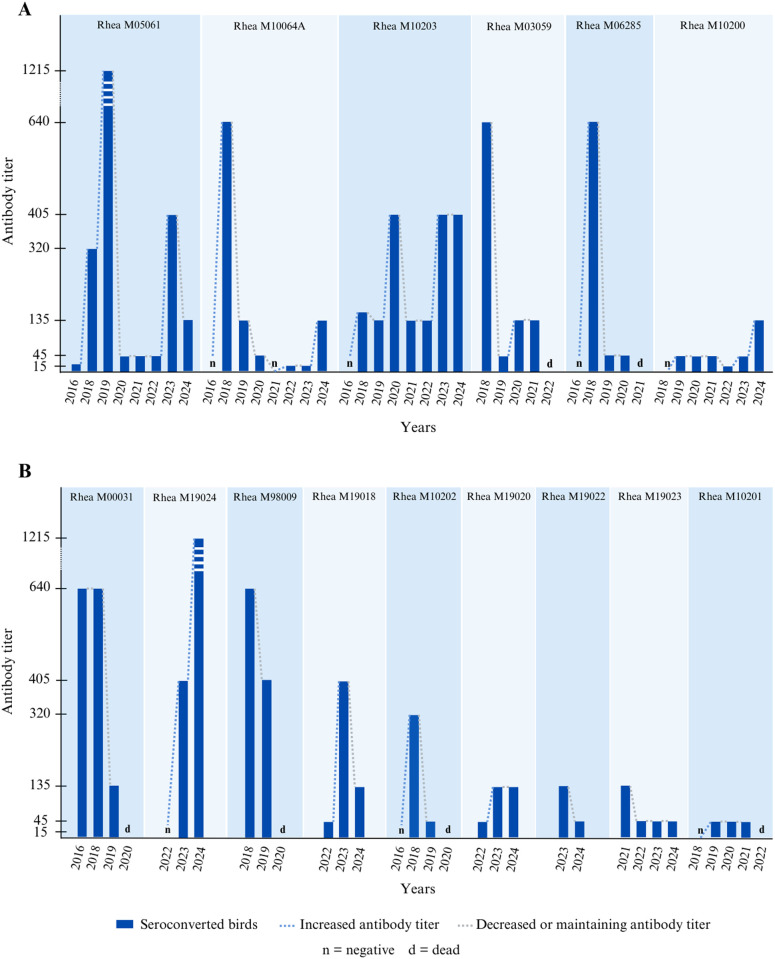
Individual rheas MNT serological results across time. Resulting of availability and death, rheas have different monitoring time spans. **A)** Rheas with the most information; B) other rheas. Figure created using canva.com.

**Fig 4:**
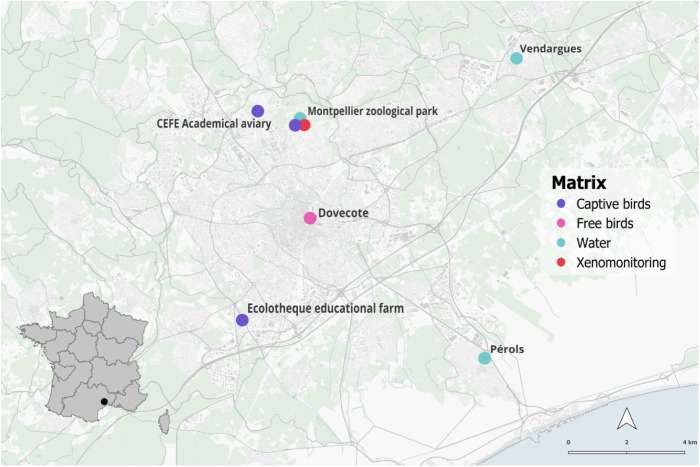
Sites, tested matrices and USUV detection in the Montpellier metropolitan area (black dot on the general map). Base map data from OpenStreetMap, via Geofabrik (https://download.geofabrik.de/), under the Open Database License (ODbL). Sea layer created by the authors. Administrative boundaries obtained from freely available open data sources.

#### 1.1. Avian and rodent samples.

Avian zoo samples were taken between 2016 and 2024, with exception of 2017 where birds were not sampled, from a total of 155 birds of 10 different species (*Rhea americana*; *Eudocimus ruber*; *Bucorvus abyssinicus*; *Dromaius novaehollandiae*; *Pavo cristatus*; *Amazonetta brasiliensis*; *Gallus gallus*; *Chauna torquata*; *Leptoptilos crumenifer*; *Ara ambiguus*). Samples from the Ecolotheque include sera from 33 birds of 7 different species (*Anas platyrhynchos*; *Cairina moschata*; *Anas platyrhynchos domesticus*; *Meleagris gallopavo*; *Anser anser domesticus*; *Gallus gallus*; *Numida meleagris*), collected only in October 2024, and blood and organs from a dead helmeted guineafowl (*Numida meleagris*) from March 2024. For these animals, 0.3 to 1.5 mL of heparinized plasma or serum were taken from the medial metatarsal vein or the brachial vein. The blood was centrifuged at 2000 G for 10 min, the serum was then separated from the clot and stored at -20°C until analysis.

Cloacal swab was taken by inserting the swab into the cloaca of the bird and then returning it to the liquid containing an RNA preservative (stored at -20°C until analysis). Complete brain and part of the liver and spleen were also taken after necropsy for a few animals of interest.

Great tits (*Parus major*) were blood-sampled throughout 2024 at the CEFE (a peri-urban area close to Montpellier zoological park), reaching 36 Dried Blood Spots (DBS, corresponding to 10–15 µL of blood deposited on Whatman Saver Cards). A total of 39 cloacal swabs were collected.

We collected two pools of pigeon droppings (*Columba livia*) 21 days after the death of three pigeons whose corpses were found in the same loft in Montpellier Center (urban area). The samples were placed in two 50 mL tubes and stored at -20°C until analysis.

Moreover, we analysed 31 DBS of black rats (*Rattus rattus*) captured on the Montpellier zoological park site in 2023 and 2024. Blood spots were collected from the heart on filter paper (Whatman), then dried for 24 hours and stored at room temperature in an individual resealable plastic bag with a desiccant pack (silicagel).

#### 1.2. Molecular xenomonitoring (MX) samples.

Mosquito trapping at Montpellier zoological park consisted in two traps: one BG-Pro trap (Biogents, Germany) connected to the electrical grid – allowing a continuous functioning between two successive pick-ups – placed next to the enclosure of the greater rheas in Montpellier zoological park, and a second BG-Pro trap on 64h-autonomy battery (Powerbank 27 000 mAh) placed inside the enclosure to directly monitor mosquito presence and potential virus circulation in close proximity to the birds. The mosquito sampling at the Ecolotheque educational farm consisted of a BG-Sentinel trap connected to the mains, placed inside the duck enclosure and their pool. All three traps were associated with a 3D-printed plastic MX adapter to collect the faeces of mosquitoes kept alive in the adapter [33]. Two types of baits were used to attract *Culicidae* species: CO2 generated by BG-CO2-Generator (Biogents) and BG-Lure-Mozzibait [34,35]. Inside the adapter, a large feeder (which is twice the volume of the regular feeder) composed of a cotton ball soaked in a 10% honey water solution was placed to maintain humidity and sustain the trapped mosquitoes alive during the whole week. At the bottom of the MX adapter, a ring of regular kitchen aluminium foil (10µm thickness) was used to collect mosquito faeces. The traps were collected every week between the 15th of April and the 23rd of October 2024 (i.e., 28 sessions) for the first trap at Montpellier zoological park, between the 23rd of July and the 8th of October (i.e., 12 sessions) for the second trap, and from the 22nd of July to the 7th of October (i.e., 12 sessions) at the Ecolotheque. In the laboratory, the mosquitos trapped in the MX adapter were firstly collected with an entomological vacuum Prockopack, frozen-killed at -20°C, sexed and identified to the genus level. The aluminium ring was collected, then provided to IAGE (Ingénierie et Analyse en Génétique Environnementale) and stored at -80°C before RT-dPCR analysis.

In addition to these routine sampling sites, two additional MX collections were conducted opportunistically in response to confirmed autochthonous arboviral cases. In Perols, 3 MX traps were deployed between July 5 and 8, 2024. In Vendargues, 3 MX traps were deployed in three periods: September 20–23, September 23–30, and September 27–30 2024. These opportunistic trappings were also supplemented with a BG-CO2 Generator (Biogents) and a BG-Lure Mozzibait.

#### 1.3. Environmental water samples.

Environmental water samples were collected from the same four sites where mosquitoes were sampled, using standardized, sterilized 1 L polypropylene bottles. The water was first homogenised at the surface and at depth, then directly recovered with the bottles. The first site was a large concrete pool (14,000 m³) within the enclosure of the greater rheas at the Montpellier zoological park. The second site was the Montpellier Ecolotheque, where a single pooled environmental water sample was generated with various samples from different avian resting areas, including small concrete pools and bathing zones used by geese, chickens, ducks, and guinea fowl. To ensure sampling homogeneity, the water column was gently mixed, incorporating both surface and deeper water layers while avoiding sediment disturbance, before collecting a 1 L targeted sample. Environmental water sampling was conducted monthly between July and September 2024. After collection, the water samples were kept in 4°C coolers and refrigerators, before analyses within 48h at IAGE laboratory. During each sampling event, after homogenization, the water physico-chemical parameters — pH, conductivity, salinity, and temperature — were measured using a HI98195 multi-parameter probe (Hanna Instruments, USA), and the turbidity was assessed through the measure of absorbance (570 nanometer), realized in laboratory with microplate spectrophotometer Thermo Scientific Multiskan SkyHigh.

Like the MX trapping, environmental water sampling was also conducted opportunistically during mosquito control operations. Hence, in addition to the routine sampling sites, two additional water collections were performed in response to confirmed autochthonous dengue cases in Perols and Vendargues. These samples were obtained from multiple *Culex pipiens* and *Aedes albopictus* larval breeding sites. In Perols, water samples were collected between July 5 and 8, 2024. In Vendargues, water samples were collected on September 20, 23, and 26, 2024.

### 2. Serological analyses

#### 2.1. ELISA test.

The presence of anti-orthoflavivirus antibodies was detected using the ID Screen Flavivirus Competition ELISA kit (IDVet, France) with sera or DBS of birds (zoo, CEFE) and rats. The plates are pre-coated with a flavivirus envelope protein. Fifty microliters of serum were added to each designated well and incubated for 1.5 hours, allowing any antibodies against orthoflavivirus in the serum to bind to the coated antigen. Afterward, 100 µL of secondary antibody is added and incubated for 30 minutes, during which it attaches to the coated antigen if antibodies are present in the serum. The plates are then washed, and 100 µL of the substrate solution is added for 15 minutes. A colour change indicates the presence of the secondary antibody, signalling a negative test result (DO > 0.788 = negative result; DO < 0.0630 = positive result). For DBS 2 punches were resuspended in a 2 mL tube with 100 µL of PBS1x/BSA/Tween 0.1% buffer and incubated overnight at 4°C, following the IDVet kit protocol. Then, 50 µL of the resuspended solution was added to the plate and incubated overnight at room temperature. After rinsing, 100 µL of conjugate was added to the plate.

#### 2.2. Microneutralization assays.

Vero E6 cell cultures were used for microneutralization tests (MNT). These cells were cultured with Dulbecco’s modified Eagle’s medium (DMEM; Sigma-Aldrich) supplemented with 10% heat-inactivated fetal bovine serum (Dutscher) and Penicillin/Streptomycin antibiotics (Gibco), kept in an incubator at 37°C and 5% CO2. The MNT was carried out in flat-bottomed 96 well microplates. 15 µL of avian serum (zoo, CEFE) was subsequently added to the first column to obtain a starting dilution of 1/5. From this well, three-fold dilutions were made by pipetting 50 µL of each well in the next, until the last column. For DBS samples, 1 punch in 50 µL of PBS1x/BSA/Tween 0.1% buffer, incubate overnight at 4°C, add the 15 µL in the plate. The sera were mixed with 50 µL of virus suspension (at 100 tissue culture infectious dose 50 (TCID50) of USUV (France2018, MT863562) incubated at 37°C for 90 min to allow neutralization of the virus by antibodies. For DBS samples, incubated overnight at 37°C. Wells were then supplemented with 100 µL of Vero E6 cell suspension (DMEM with 2% foetal bovine serum) and incubated 3–6 days at 37°C and under 5% CO2. After this time, microplates were read under the microscope to determine the presence or absence of cytopathogenic effect (ECP) in samples and in positive and negative sera controls. Each sample was assigned a titer that was the reciprocal of the dilution in that well. Sera that showed neutralization at dilutions ≥ 1:15 were considered positive. Duplicates were performed to confirm positive results. For the samples collected between 2016 and 2019, MNT assays were conducted under identical conditions to verify the absence of anti-WNV antibodies using WNV lineage 2 (strain MT863560).

### 3. Molecular analyses

#### 3.1. RNA extraction, RT-qPCR and sequencing.

RNA extractions were carried out of organs, cloacal swabs, sera, droppings and DBS using the QIAamp viral RNA mini kit (Qiagen), following the manufacturer’s protocol. For DBS sample, one punch per card was individually rehydrated in 175 µL of PBS1x/BSA/Tween 0.1% buffer and incubated overnight under agitation at 4 °C. A volume of 140 µL of the resulting suspension was then used for RNA extraction according to the kit protocol. For organs and droppings, samples were directly ground in 140 µL of PBS1x, and the entire lysate was used for extraction. Subsequent extraction steps were performed according to the QIAamp Viral RNA Mini Kit protocol. All RNA samples were eluted in 50 µL of elution buffer, as recommended. RNA quantity and purity were assessed by measuring absorbance using a Nanodrop spectrophotometer. Reverse transcription (RT) was then performed on 500 ng of total RNA using the LunaScript RT SuperMix Kit (New England BioLabs).

Quantitative PCR (qPCR) was performed using the SYBR Green I Master mix (Roche) on a LightCycler 480 instrument (Roche), with the following primers targeting the Usutu virus genome: forward primer 5′-AACAGACGGTGATGCGAACT-3′ and reverse primer 5′-TACAGCTTCGGAAACGGCTT-3′ [36]. Thermal cycling conditions included an initial activation at 95 °C for 10 minutes, followed by 45 cycles of amplification at 95 °C for 15 seconds, 60 °C for 15 seconds, and 72 °C for 15 seconds. A melting curve analysis was subsequently performed with the following steps: 95 °C for 5 seconds, 65 °C for 1 minute, and a continuous increase to 97 °C.

To validate selected results, a one-step TaqMan RT-qPCR assay was also conducted using RNA extracted from the same samples. As this was a one-step assay, reverse transcription and amplification were conducted in a single reaction. A volume of 5 μL of each RNA extract was directly used as input. Reactions were carried out using the SuperScript IV One-Step RT-PCR System (Invitrogen) with primers and probe targeting the NS5 region of the Usutu virus genome: forward primer 5′-AAAAATGTACGCGGATGACACA-3′, reverse primer 5′-TTTGGCCTCGTTGTCAAGATC-3′, and probe 5′FAM-CGGCTGGGACACCCGGATAACC-3′TAMRA [37]. Thermal cycling was conducted on a LightCycler 480 instrument (Roche) under the following conditions: reverse transcription at 50°C for 15 minutes; enzyme activation at 95°C for 2 minutes; followed by 50 amplification cycles consisting of denaturation at 95°C for 15 seconds and combined annealing/extension at 60°C for 1 minute. Fluorescence data were collected at the end of each extension step. A virus-derived RNA extract from a viral culture was included in each run as a positive control.

To confirm some samples, amplicons obtained at the end of the RT-qPCR were purified using the Gel and PCR Clean-up kit (NucleoSpin) and then sent for sequencing. Sanger sequencing was carried out by GENEWIZ from Azenta Life Sciences starting from 20 µl of purified amplicons, using one of the primers employed for amplification.

#### 3.2. Environmental water analyses and xenomonitoring analyses.

***3.2.1. RNA/DNA extraction for water analyses***. Extractions of total DNA/RNA for dPCR analyses were performed by IAGE Company following the patented method [38] with an optimized pre-treatment. Briefly, the water samples were kept at 4°C after sampling until nucleic acid extraction with volumes comprised between 100 mL and 1 L. The Environmental water sample were then thoroughly mixed, 15 mL were taken from each homogenized sample and centrifuged for 15 min at 3234 g at 4°C. For each sample, pellet was conserved on ice until further treatment. Supernatant was recovered and applied to an Amicon Ultra-15 Centrifugal Filter Unit (cut-off: 10 kDa, Cat. No. UFC901096, Merck Millipore, Germany) previously hydrated with 2,5 mL of MQ water and spun for 10 min at 3234 g, at 4°C. The tube was then centrifuged for 35 minutes at 3234 g, at 4°C. If the resulting volume was inferior to 500 µL, the tube was spin again for 10 minutes at 3234 g, at 4°C. The resulting volume was used to resuspend the corresponding pellet for each sample. Then, 400 µL of Nucleo Protect VET Buffer (Cat. No. 740750, Macherey Nagel, Germany) was added before grinding with two 3 mm stainless steel beads on a Geno/Grinder instrument (Spex Sample Prep LLC, USA) for 15 s at 1500 cpm, thrice. Extractions were then performed using the Indimag Pathogen kit (Cat. No. SP947457, Indical Bioscience, Germany) following manufacturer’s instructions.

***3.2.2. RNA/DNA extraction for xenomonitoring analyses.*** The aluminium foil from the MX adapter was coiled and placed individually in 50 mL plastic tubes (Greiner Bio-one, Cat. No.227261) before being soaked in a 1.2 mL VXL lysis buffer (Cat. No.1069974, Qiagen, Germany) and vortexed for 5 min. The supernatant was recovered in 1.5 mL tubes and 200 µL were used for DNA/RNA extraction using the Indimag Pathogen Kit (Cat. No. SP947457, Indical bioscience, Germany) following manufacturer’s instructions. Then, the sample pre-treatment for total nucleic acid extraction followed the same protocol as the water sample analyses.

***3.2.3. RT-dPCR***. Digital-PCR reactions were conducted using a simplex assay developed by IAGE on a QIAcuity Eight Plateform System (Qiagen, Germany), using the QIAcuity One-Step Viral RT-PCR Kit (Cat. No. 210212) and QIAcuity Nanoplate 26 K 24-wells (Cat. No. 250001). The dPCR detection system was adapted from [39] with primers USUTU F (5′-CGTTCTCGACTTTGACTA-3′) and USUTU R (5′-GCTAGTAGTAGTTCTTATGGA-3′) optimized for enhanced specificity to USUV by the IAGE company (https://www.iage-france.com). The dPCR reaction mixtures were prepared in a preplate as follows. For each reaction 10 µL of 4 × One-Step Viral RT-PCR Master Mix, 0.4 μL of 100 × Multiplex Reverse Transcription Mix, 1 μL of the primers/probes mix (final concentration 0.45 µM of both primers and 0.125 µM of probe), and 2 µL of DNA extract (for both xenomonitoring assays and water samples) were combined with H2O up to 40 µL final reaction volume. Reaction mixtures were transferred into a QIAcuity Nanoplate and the plate was loaded onto the QIAcuity Eight instrument, a fully automated system. The workflow included (i) priming and rolling step to generate and isolate the chamber partitions (26,000 partitions), (ii) amplification step under the following cycling protocol: 50 °C for 40 min for reverse transcription, 95 °C for 2 min for enzyme activation, 95 °C for 5 s for denaturation, and 58 °C for 60 s for annealing/extension for 40 cycles; and (iii) imaging step by reading fluorescence emission after excitation of the probe at the appropriate wavelength. Data were analysed using the QIAcuity Software suite V3.1.0.0.

### 4. Statistical analyses

The overall prevalence was calculated using two-sided exact binomial 95% confidence intervals based on the Wald method (95% CI). Birds included in the incidence analyses were those with at least one follow-up sample over one year, and initially seronegative. The overall incidence of *Orthoflavivirus* infection was calculated for each species as the number of positive serological cases divided by the total bird-years. As the samples of zoo birds are taken in October every year, *Orthoflavivirus* infections were assumed to have occurred in the year (October to October) of the first positive serological test. *Orthoflavivirus*-free birds were censored at the date of their last negative test. Results are presented in 10 bird-years with a 95% Poisson confidence interval. All statistical analyses were performed using R software (R Core Team, 2024) within the RStudio environment (RStudio Team, version 4.4.2, 2024).

## Results

### 1. Circulation of USUV in Montpellier zoological park: Monitoring in captive exotic Avifauna (2016–2024), Mosquitoes, and Water (2024).

#### 1.1. Captive exotic Avifauna.

Analyses were carried out on 155 specimens of 10 different avian species across a period running from 2016 to 2024 (Fig 1). No samples were available in 2017.

Using ELISA serological tests, we identified antibodies against *Orthoflaviviruses* in 41 out of 155 birds (26.5%, CI95% [19.5-33.4]), and 15 of the 41 were greater rheas (*Rhea americana)*, (36.5%, CI95% [21.8-51.3]; Fig 2, S1 Table). The *Orthoflavivirus* incidence rate, used here to calculate the rate of seroconverted birds per species over a given monitoring period (per 10 bird-years), was determined based on 127 individuals from 9 different species. The highest incidence rate was observed in *Rhea americana* 3.44 [1.72;6.15] (other birds: *Bucorvus abyssinicus* 1.43 [0.29;4.17]; *Pavo cristatus* 0.71 [0.09;2.58]; *Ara ambiguus* 0.62 [0.02;3.48]; *Gallus gallus* 0.59 [0.01;3.28]; *Amazonetta brasiliensis* 0.57 [0.07;2.06]; *Eudocimus ruber* 0.43 [0.18;0.84]). We therefore observed the seroconversion of many birds throughout our study, with incidence data indicating that, for most of them, this seroconversion occurred between 2016 and 2019 but has also continued up to 2024. Notably, new animals seroconvert each year.

Of the 41 ELISA-positive zoo birds, 37 could be tested for USUV-specific antibodies by virus-specific microneutralization test (MNT), as we no longer had material for the other 4 (*Eudocimus ruber*). Of these 37 birds tested by MNT USUV, 28 tested positive for the presence of antibodies against USUV (75.6%, CI95% [61.8-89.5]), including the 15 greater rheas that tested positive by ELISA (53.5%, CI95% [35.1-72]), representing the total number of individuals tested (Fig 3, S2 Table). Since 2021, only one rhea has not developed neutralizing antibodies against USUV. In greater rheas, we observed different patterns of immunity maintenance. Some individuals exhibited a peak in neutralizing antibody titers in one year, followed by a significant decline, while others experienced fluctuations with alternating increases and decreases in antibody levels over time. Additionally, some individuals showed an increase in antibody titers after a previous decline, suggesting regular reinfections and, consequently, continuous circulation of USUV within the Montpellier Zoo. Notably, microneutralization assays for WNV performed on samples collected during the early years (2016–2019) of the study did not reveal any WNV positive cases among the avian species monitored throughout the study.

In parallel with our serological analyses, we extracted and analysed organs, blood samples, and cloacal swabs by RT-qPCR mainly from 2024, along with some organs and cloacal swabs dating from 2020 to 2023. From bird sera and cloacal swabs collected in August and October 2024, we detected the presence of USUV RNA in 11 of the 26 birds tested (42.31%, CI95% [23.32-61.30]), including 5 positive greater rheas out of 15 (33.3%, CI95% [9.48-57.19]) (Table 1, S3 Table). We were unable to obtain a complete sequence of our samples due to the limited amount of material available. Nevertheless, several amplicons were sequenced, confirming the positivity of the samples with sequences likely related to the Africa 3 lineage strains (≈98% identity) (S1 Appendix).

**Table 1:** *+Molecular analyses of rheas by RT-qPCR.

Rheas tested		Date of sample	Nature of sample	Presence (+) or absence (-) of USUV viral RNA
**Lunaret zoological park**	Rhea M19019	01/2020 (dead)	Brain	(-)
	Rhea M98009	10/2020 (dead)	Brain	(-)
	Rhea M06285	07/2021 (dead)	Brain	(-)
	Rhea M10201	11/2021 (dead)	Brain	(-)
	Rhea M03059	07/2022 (dead)	Brain	(-)
	Rhea M19018	10/2023	Cloaca	(-)
		08/2024	Serum	**(+)**
		09/2024 (dead)	Serum	(-)
			Brain	**(+)**
			Spleen	**(+)**
	Rhea M19023	10/2023	Cloaca	(-)
	Rhea M10064A	10/2023	Cloaca	(-)
		08/2024	Serum	(-)
		10/2024	Cloaca	**(+)**
	Rhea M10200	10/2023	Cloaca	(-)
		08-10/2024	Serum and cloaca	(-)
	Rhea M10203	10/2023	Cloaca	(-)
		08-10/2024	Serum and cloaca	(-)
	Rhea M19024	10/2023	Cloaca	(-)
		08/2024	Serum	(-)
		10/2024	Cloaca	**(+)**
	Rhea M19020	10/2023	Cloaca	(-)
		08/2024	Serum	**(+)**
		10/2024	Cloaca	(-)
	Rhea M19021	10/2023	Cloaca	(-)
		08/2024	Cloaca	(-)
	Rhea M19022	10/2023	Cloaca	(-)
		08-10/2024	Serum and cloaca	(-)
	Rhea M05061	10/2023	Cloaca	(-)
		08/2024	Serum	**(+)**

(+) = Usutu viral RNA detected; (-) = no Usutu viral RNA detected. No comparative assessment of viral load was performed.

#### 1.2 USUV infections in mosquitoes’ faeces.

Since these greater rheas appear to be particularly susceptible to USUV infection, we set up a xenomonitoring mosquito trap near their enclosure from May to September 2024 to determine whether we could detect USUV viral RNA. Only mosquito faeces collected from the trap were analysed, not the mosquitoes themselves. We detected a positive signal in September, indicating the presence of the virus in mosquitoes near these animals. A total of 30 *Aedes* (24 females, 6 males), 208 *Culex* (148 females, 60 males) and 2 *Culiseta* (1 of each sex) were captured inside the enclosure during the 28-sessions course of the sampling, and 13 *Aedes* (12 females, 1 male), 360 *Culex* (239 females, 121 males) and 32 *Culiseta* (29 females, 3 males) outside of it, in 12 sessions (S2 Appendix). The faeces of these mosquitoes were analysed, and USUV was detected in samples collected during the week of September 3–10, 2024 for the traps inside the rheas’ enclosure. In this trap, a total of 32 female and 28 male *Culex* spp. mosquitoes were captured, along with eight female and one male *Aedes albopictus*.

#### 1.3. USUV detection in rheas’ pool water.

The particularly high circulation of USUV among greater rheas led us to question the possibility of detecting this virus in the water of their concrete pool, which was emptied and cleaned monthly. Based on the hypothesis that these animals might shed the virus in their faeces, we collected water samples from the pool from July to September 2024, during the period when arboviral circulation is known to be most intense in the region. Interestingly, we detected a positive signal in the pool water in July, before detection in other matrices such as mosquitoes, highlighting the relevance of this matrix for the viral detection of the USUV (Table 2).

**Table 2:** Molecular table analyses of the zoo pool of the greater rheas by dPCR with physicochemical parameters. The limit of detection (LOD) for the environmental water sample is: 5.97 × 10⁴ copies/L.

Physico-chemical water parameters			USUV RNA detected by dPCR
**July**	Temperature	24.58°C	**(+)** **6.23.10^4 copies per litre**
	pH	7.20	
	Salinity	0.31 PSU	
	Absorbance (570 nm)	0.053	
	Oxydo Reduction Potential	235 mV	
	Conductivity	643 µS/cm	
	Total Dissolved Solids	0.321 g/L	
**August**	Temperature	23.38°C	ND
	pH	7.69	
	Salinity	0.34 PSU	
	Absorbance (570 nm)	0.036	
	Oxydo Reduction Potential	73.2 mV	
	Conductivity	701 µS/cm	
	Total Dissolved Solids	0.350 g/L	
**September**	Temperature	19.79°C	ND
	pH	7.80	
	Salinity	0.27 PSU	
	Absorbance (570 nm)	0.044	
	Oxydo Reduction Potential	56.7 mV	
	Conductivity	495 µS/cm	
	Total Dissolved Solids	0.275 g/L	

USUV = Usutu virus; ND = not detected by dPCR; (+) = detected by dPCR with concentration; PSU = practical salinity unit. Altogether these results from the Montpellier zoological park provide a comprehensive view of the possibility of detecting the Usutu virus in various matrices within a specific location. The detection of a significant number of seroconversions in certain avian species, along with the molecular identification of the virus in greater rheas, nearby mosquitoes, and environmental water sampled in their enclosure, underscores the potential for a global monitoring of this virus across both environmental and biological samples.

### 2. Circulation of USUV in the surrounding of Montpellier

Given the active circulation of the Usutu virus over the years demonstrated at the Montpellier zoological park, we expanded our study in 2024 to other sites and different types of samples. Thus, we included the Ecolotheque, an educational farm which hosts various bird species, as well as the CEFE, which has an aviary with great tits, species known to be susceptible to USUV infection. We also conducted a targeted analysis of a pigeon loft located in the heart of the city of Montpellier, examining pigeon droppings following a series of unexplained deaths. Additionally, we studied two other sites within the Montpellier metropolitan area to explore the possibility of detecting the presence of the virus in water at locations with a lower concentration of birds compared to the zoo (Fig 4).

Of 33 bird sera collected in October 2024 and analysed by pan-flavivirus ELISA from the Ecolotheque (2 mallard ducks *(Anas platyrhynchos)*, 5 Muscovy ducks *(Cairina moschata)*, 2 Indian runner ducks *(Anas platyrhynchos domesticus),* 2 wild turkeys *(Meleagris gallopavo),* 4 domestic geese *(Anser anser domesticus),* 16 red junglefowl *(Gallus gallus)*, 2 helmeted guineafowl *(Numida meleagris)*), 7 individuals were positive for the presence of anti-orthoflaviviruses antibodies (1 *Anas platyrhynchos*; 1 *Cairina moschata*; 1 *Meleagris gallopavo*; 4 *Anser anser domesticus*). Of these 7 ELISA-positive samples, 2 were found to be positive for the presence of antibodies against USUV by MNT (1 *Meleagris gallopavo*; 1 *Anser anser domesticus*) (Table 3).

**Table 3:** ELISA and MNT serological analyses on birds from the Ecolotheque and CEFE tits.

Avian species (*latin name*)			2024 seroconverted individuals detected by orthoflavivirus ELISA	USUV-MNT antibody titer (for ELISA positive individuals)	
**Ecolotheque**	**Mallard duck (n = 2)** (*Anas platyrhynchos*)		**1 (+) ELISA**	(-)	
	**Muscovy duck (n = 5)** (*Cairina moschata*)		**1 (+) ELISA**	(-)	
	**Indian runner duck (n = 2)** (*Anas platyrhynchos domesticus*)		(-)	/	
	**Wild turkey (n = 2)** (*Meleagris gallopavo*)		**1 (+) ELISA**	**1 (+) MNT**	**Titer 45**
	**Domestic geese (n = 4)** (*Anser anser domesticus*)		**4 (+) ELISA**	**1 (+) MNT**	**Titer 135**
				3 (-)	**/**
	**Red junglefowl (n = 17)** (*Gallus gallus*)		(-)	/	
	**Helmeted guineafowl (n = 2)** (*Numida meleagris*)		(-)	/	
**CEFE**	**Great tits (n = 47)** (*Parus major*)	DBS samples	**1 (+) ELISA**	(-)	

(+) = presence of antibodies in sera; (-) = sample tested but absence of antibodies in sera;/ = sample not tested because of negative ELISA tests. CEFE = Center for Functional and Evolutionary Ecology.

Following molecular analyses, seven birds were tested positive for the presence of USUV RNA. The virus RNA was detected in cloacal swabs from one helmeted guineafowl (*Numida meleagris*), one Muscovy duck (*Cairina moschata*), one wild turkey (*Meleagris gallopavo*), and four domestic geese (*Anser anser domesticus*) (Table 4). We also detected USUV RNA in the liver and brain of a helmeted guineafowl (*Numida meleagris*) that died in March 2024, possibly due to USUV infection. (20%, CI95% [6.75-33.25]). 33 birds were also tested on their serum, but none were identified as positive (red junglefowl (*Gallus gallus*); Muscovy duck (*Cairina moschata*); mallard duck (*Anas platyrhynchos*); Indian runner duck (*Anas platyrhynchos domesticus)*; domestic geese (*Anser anser domesticus*); wild turkey (*Meleagris gallopavo*); helmeted guineafowl (*Numida meleagris*)). These USUV detections at the Ecolotheque indicate an extended circulation of USUV in the Montpellier metropolitan area, given that this site is located 9 km from the zoological park (Fig 4).

**Table 4:** Molecular analyses of Ecolotheque, CEFE and pigeon loft (3M) by RT-qPCR.

Birds tested		Date of sample	Nature of sample	Presence (+) or absence (-) of USUV viral RNA
**Ecolotheque**	**Helmeted guineafowl (n = 1)** (*Numida meleagris*)	03/2024 (dead)	Brain, Liver	**(+)**
	**Domestic geese (n = 4)** (*Anser anser domesticus*)	10/2024	Cloaca	**(+)**
	**Muscovy duck (n = 1)** (*Cairina moschata*)	10/2024	Cloaca	**(+)**
	**Wild turkey (n = 2)** (*Meleagris gallopavo*)	10/2024	Cloaca	**(+)**
**CEFE**	**Great tits (n = 26)** (*Parus major*) Z28, Z89, Z57, Z53, Z43, Z34, Z30, Z19, Z31, Z15, Z91, Z38, Z45, Z18, Z52, Z00, Z86, Z42, Z28, Z20, Z90, Z16, Z87, Z04, Z95, Z93	10/2024	Cloaca	(-)
	**Great tits (n = 7)** (*Parus major*) Z49, Z02, Z01, Z27, Z23, Z98, Z92	10/2024	Cloaca	**(+)**
			DBS	(-)
	**Great tits (n = 2)** (*Parus major*) Z54, Z13	10/2024	Cloaca	**(+)**
			DBS	**(+)**
**3M**	**Pigeons (n = 60)** (*Columba livia*) (2 pools of n droppings)	09/2024	Droppings pool 1	(-)
			Droppings pool 2	**(+)**

(+) = Usutu viral RNA detected; (-) = no Usutu viral RNA detected. 3M = Montpellier Méditerranée Métropole, CEFE = Center for Functional and Evolutionary Ecology.

We also analysed DBS and cloaca swabs from great tits. We detected a single positive great tit in the pan-flavivirus ELISA test among the 36 tits tested for serological analyses. However, this tit tested negative in the MNT for anti-USUV antibodies (Table 3). As for the exotic avifauna from the zoological park, we also aimed to deepen our analyses with molecular tests. We analysed 35 cloaca samples from CEFE tits and detected fragments of USUV viral RNA in 9 of them (25%, CI 95% [10.86-39.14]). Since USUV is naturally present in the blood of infected species, we attempted to detect viral RNA fragments in the DBS samples corresponding to individuals that were positive in cloacal swabs. We identified 2 positive DBS samples out of 8 tested (with insufficient material remaining for analysis for one individual) (Table 4).

We therefore attempted to deepen these analyses using pigeon droppings (*Columba livia*), an original sampling matrix. One of the two pools tested positive for the presence of USUV viral RNA fragments, suggesting the circulation of USUV within this pigeon loft (Table 4).

The detection of USUV in the environmental water sample from the greater rhea’s enclosure demonstrated that this detection strategy is effective for sites with high viral circulation. Based on this, we decided to extend our analyses to other locations with no known history of USUV circulation. We therefore tested several environmental water sampling locations around Montpellier for Usutu virus RNA. Out of 3 locations analysed around Montpellier, we detected the presence of Usutu virus RNA by dPCR on 2 of them: in Perols in July 2024 (8.35x10^4^ copies/L) and in Vendargues in September 2024 (5.88x10^4^ copies/L) (Fig 4.). These 2 positive detections on environmental water samples provide further support for implementing large scale environmental surveillance based on those analyses.

At the Ecolotheque, 447 *Aedes* (301 females, 146 males), 204 *Culex* (148 females, 56 males) and 19 *Culiseta* (all females) were captured in 12 capture sessions. Finally, during the opportunistic trapping, 73 *Aedes* (56 females, 15 males), 4 *Culex* (196 females, 1 males) and 2 *Culiseta* (both males) were captured in Vendargues, while 21 *Aedes* (11 females, 10 males), 12 *Culex* (6 of each sex) and 1 male *Culiseta* were captured in Perols (S2 Appendix). Mosquito faeces from all these traps returned negative to USUV.

## Discussion

In this study, we conducted a long-term monitoring of Usutu virus in zoo animals over a 7-year follow-up in several matrices, including exotic and local birds, environmental water, and mosquito faeces. Our results show widespread circulation of the virus within and around the city of Montpellier, in an urban context.

From the zoo investigation, we identified species particularly susceptible to USUV infection. In particular, nearly all greater rheas we monitored showed seroconversion over the years, which was less pronounced in other birds, although regular seroconversions were also observed in other species such as blue peafowl (*Pavo cristatus)*, emu *(Dromaius novaehollandiae)* or Abyssinian ground-hornbill (*Bucorvus abyssinicus).* The particular sensitivity of the greater rheas to USUV infection could stem from their large size (1.50 meters), which may increase mosquito attraction due to larger surface area and number of bites, as well as their proximity to several water sources, which could serve as a larval habitat, promoting the prevalence of *Culex* mosquitoes. Greater rheas may also have an immune system particularly sensitive to USUV compared to other birds. Although not being a native species in Europe, greater rheas imported into parks or zoological reserves for a few decades, have a 20-year lifespan and showed sensitivity to USUV, making them a good sentinel species for monitoring immunity against USUV. An example of successful sentinel use is the routine monitoring of captive species found dead at the Bronx zoo in New York in 1999, including American crows (*Corvus brachyrhynchos*), American flamingos (*Phoenicopterus ruber*), golden pheasants (*Chrysolophus pictus*), barred owls (*Strix varia*), and bald eagles (*Haliaeetus leucocephalus*) which led to the discovery of WNV as a cause of human morbidity and mortality in the United States [40]. Previous reports have documented USUV infection in birds in zoological parks. In 2001, five great grey owls (*Strix nebulosa*) died from USUV infection at a zoo in Vienna [9]. Then, in 2006 at the Zurich zoo, both wild and captive avian species of *Passeriformes* (thrushes and sparrows) and *Strigiformes* (owls) orders experienced a significant mortality, and USUV was identified in a variety of species, including African marabou stork (*Leptoptilos crumenifer*), ruddy shelduck (*Tadorna ferruginea*), red-breasted goose (*Branta ruficollis*), Humboldt penguin (*Spheniscus humboldti*), laughing kookaburra (*Dacelo novaeguineae*), steamer duck (*Tachyeres pteneres*), and domestic chicken (*Gallus gallus domesticus*) [41]. Additionally, as part of the same study, the USUV was also identified in six American flamingos (*Phoenicopterus ruber*) at Basel zoo and in a snowy owl (*Bubo scandiacus*) at Vienna zoo. Furthermore, various mammals in zoological parks can host susceptible species, also demonstrated by a study conducted in Spain, which found several mammals with antibodies against USUV, similar to another study we carried out at the Montpellier zoological park [20,42].

Zoological parks typically house a wide range of captive species, including birds and mammals in direct or indirect contact with mosquitoes and wild birds as well as other animals such as rodents which can be potential carriers of USUV. In light of our results on the presence of USUV at the Montpellier zoological park and USUV detection in rodents [27], we explored the possibility of detecting seroconversion in DBS samples from rats captured within the zoo. However, we were unable to detect antibodies against *Orthoflaviviruses* in these samples, possibly due to the small sample size tested. A broader study using serum could be valuable to identify potential seroconversion or even to detect the USUV genome at the molecular level in rodents from the region. This would help determine whether these animals could potentially serve as secondary reservoirs of the virus in our study area.

It is interesting to note that in our study, zoo animals are regularly exposed to infections, as evidenced by the increased levels of neutralizing antibodies directed against USUV. The significant decrease in neutralizing antibody levels from one year to the next also indicates that immunity is transient in these exotic birds, allowing for possible reinfections, as suggested by year-to-year increases in USUV-specific antibody titers observed in our study. It should be noted that, while one animal died with USUV virus detected in the brain and liver, we could not directly link its death to the infection, possibly due to other underlying pathologies. The majority of USUV infections within the zoo are silent, with no particular symptoms detected. A small proportion of the sera positive in ELISA lacked neutralizing functional antibodies against USUV, possibly due to the generally lower sensitivity of viral neutralization assays compared to ELISA tests, including the MNT. Additionally, functional and binding antibodies may decrease at different rates, which could explain ELISA-positive individuals lacking detectable neutralizing antibodies. Another possible explanation is that birds negative for the USUV MNT may have been infected with other arboviruses (e.g., WNV or Bagaza virus). It is worth noting that we did not detect any WNV infection in these animals during WNV serum neutralization tests conducted in the first years of the study.

Monitoring viral circulation is challenging, especially for viruses with complex transmission cycles. One major advantage of wild animals for monitoring arbovirus circulation like WNV and USUV is their ability to roam freely over vast areas, particularly in the case of birds. This mobility increases the probability of exposure to infected mosquitoes and spending time in regions where arboviruses are present. However, when these animals are recaptured, researchers are unable to trace their previous movements or pinpoint the exact enzootic focus. In contrast, captive species have their movements restricted, controlled, and well-documented, and allow repeated sampling over time. Therefore, typically housing a wide range of captive species - including birds and mammals - and often located near or within urban areas like the Montpellier zoological park, zoos can provide valuable information on the urban circulation of certain viruses, including USUV. Since captive animals are regularly monitored by veterinarians, their samples collected during routine procedures or through biobanking, are readily available for long-term analyses and allow for both retrospective and prospective studies, helping to identify emerging pathogens and track the epizootiological situation over the long term. Thus, studying the prevalence or seroprevalence of USUV in captive avian populations offers an effective way to monitor its circulation.

Expanding our study to additional sites within the Montpellier metropolitan area confirmed the urban circulation of USUV in other locations. We detected neutralizing antibodies against USUV and viral RNA in cloacal samples of a wild turkey and several domestic geese, and USUV RNA in a helmeted guineafowl that died in March 2024. This early-season identification raises the question of implementing an earlier surveillance system, as the current one only operates from May to November in France. This could be particularly relevant given the impacts of climate change as rising temperatures and altered precipitation patterns can extend the mosquito breeding season, accelerate viral replication within mosquitoes, and expand the geographic range of competent vectors. Simultaneously, shifts in bird migration routes and timing may increase contact between susceptible hosts and infected mosquitoes, thereby influencing transmission dynamics.

Our results on captive great tits, known to be sensitive to USUV infection, confirm the active circulation of USUV within our study area, evidenced by the identification of viral RNA in 9 of their cloacal samples. Seroprevalence and molecular study on the blood of these animals was conducted using DBS rather than serum. While it may reduce detection sensitivity compared to cloacal swabs, as the amount of serum available in DBS is lower, this approach minimizes blood sampling, facilitates fieldwork, and ensures easier sample preservation. Remarkably, we also detected viral RNA in pigeon droppings, following unexplained deaths in a pigeon loft in downtown Montpellier, highlighting the potential of non-invasive methods for identifying USUV presence in avian populations. It has previously been shown experimentally that WNV could be detected in the faeces of infected animals [43]. Our study shows the possibility of detecting USUV in cloacal swabs and bird droppings. This last approach is promising because detecting the virus in excrement is completely non-invasive for the animal, which could reduce the need for capture.

However, the use of faecal samples, whether from birds or mosquitoes, as well as other environmental matrices (such as water samples), presents clear limitations. These include lower RNA quantity and quality, degradation due to environmental exposure (e.g., UV light, temperature fluctuations, microbial activity), and the frequent presence of PCR inhibitors such as bile salts, uric acid, or plant-derived compounds. In our study, these limitations restricted us to generating only short amplicons which, while sufficient to confirm the presence of USUV and tentatively assign the strain to a major lineage, were inadequate for conducting detailed phylogenetic or phylogeographic analyses. The absence of longer, more informative genome fragments limit the ability to assess viral evolution in fine spatial and temporal frameworks. Thus, the trade-off between non-invasiveness and depth of retrievable molecular information must be carefully weighed when devising arbovirus surveillance strategies. In certain contexts, such as early detection, mapping a wide distribution or studies in conservation-sensitive areas, non-invasive approaches may be an attractive compromise. However, where detailed evolutionary or epidemiological information is required, complementary sampling strategies, possibly combining serum or organ analysis from birds with non-invasive methods, should be considered to overcome the inherent limitations of non-invasive samples.

A major challenge in studying USUV pathogenesis is the high diversity of lineages, which can co-circulate across space and time. These lineages appear to differ in virulence in both humans and birds, highlighting the need for further research to better understand their distribution and impact on animal and human health [1]. Although most strains currently circulating in Europe belong to the European lineages, African lineages continue to emerge on the continent. Notably, sequences obtained from PCR amplicons in our study suggest the presence of strains related to the African 3 lineage, consistent with previous detections reported in *Culex pipiens* in Montpellier and Camargue areas in 2015, 2018 and 2020 [29,30]. Additionally, the Africa 2 lineage was detected in the first French documented human USUV case in Montpellier, demonstrating the circulation of both lineages in the Occitanie region [32]. Montpellier is located near the Camargue, a wetland rich in biodiversity, hosting a wide variety of wild bird species, including migratory birds, as well as diverse mosquito populations. These conditions create a favourable environment for USUV transmission and required species sentinel to monitor the circulation.

It has been shown that arbovirus like WNV can also be excreted by mosquitoes [44]. The implementation of a non-invasive molecular xenomonitoring approach that uses trapped mosquito excreta allowed us to detect USUV at the Montpellier zoological park and in environmental water analysis. Indeed, once excreted by birds, mosquitoes or humans, arboviruses can be found in faeces and environmental water. Detecting arboviruses in wastewater near urban areas is already recognized as Wastewater-Based Surveillance [45–47]. Our results question the possibility of detecting USUV in environmental water near areas with high avian and vector density, such as the zoo and in areas close to human habitation. We chose digital PCR over RT-qPCR to detect USUV in water for its superior tolerance toward polymerase inhibitors commonly found in water [48]. Interestingly, we showed for the first time the possibility of detecting USUV in stagnant environmental water. This technique is a promising cost-effective alternative for large-scale studies compared to current approaches that require vector and avian analyses [45,46]. Expanding this approach to other urban, peri-urban, rural, and wetland sites would help validate its broader applicability. It would also allow evaluation of prevalence in other areas, and comparison, and compare its efficiency with other monitoring approaches currently in place.

In conclusion, our study highlights the established urban circulation of USUV in a major city in southern France, raising concerns about the potential risks of avian circulation for human health. It also emphasizes the importance of developing surveillance programs to better prevent, detect earlier, and monitor the circulation of arboviruses in urban areas, as well as identifying the avian or non-avian species that may contribute to maintaining this virus in urban environments. We thus emphasize that a One Health approach is both highly relevant and essential for studying USUV, particularly by leveraging scalable, non-invasive surveillance methods, such as monitoring viral excretion in bird droppings and environmental water samples, which enable large-scale ecosystem monitoring while minimizing animal disturbance. This integrated strategy facilitates early detection and a comprehensive understanding of virus circulation across human, animal, and environmental interfaces.

## Supporting information

S1 Table Pan-orthoflavi ELISA of zoo birds from 2016 to 2024. x/ n = x individuals positives to ELISA pan-flavi tests/ total of individuals tested on the year concerned;/ = sample not tested. (TIF)

S2 Table MNT titers of zoo birds positive for ELISA tests from 2016 to 2024. (+) = presence of anti-USUV antibodies in sera; (-) = sample tested but absence of anti-USUV antibodies in serum;/ = sample not tested.
(TIF)

S3 Table RT-qPCR molecular analyses on additional zoo birds. (+) = Usutu viral RNA detected; (-) = no Usutu viral RNA detected.
(TIF)

S1 Appendix Phylogram demonstrating the genetic relationships among USUV strains. USUV sequences are shown with their country of isolation, year of isolation, and GenBank accession numbers. Nucleotide sequences were aligned using MUSCLE software (https://www.ebi.ac.uk/Tools/msa/muscle/). The phylogenetic tree was constructed using the maximum-likelihood method in MEGA 12 software (https://www.megasoftware.net/). An asterisk (*) denotes sequences derived from our study. The scale bar represents nucleotide substitutions per site.
(TIFF)

S2 Appendix Number of captured mosquitoes, by species and sex, (a) in the Montpellier Zoo during the year 2024, inside and outside of the Rheas’ enclosure, and (b) during the opportunistic trappings in Perols (July 2024) and Vendargues (September 2024). (TIFF)
