# Supplementary figures and images for: Tracking the urban spread of Usutu virus in southern France: Detection across biological and environmental matrices
Source: PLoS Negl Trop Dis. 2025 Sep 2;19(9):e0013506. doi: 10.1371/journal.pntd.0013506 (PMC12419640; doi:10.1371/journal.pntd.0013506)

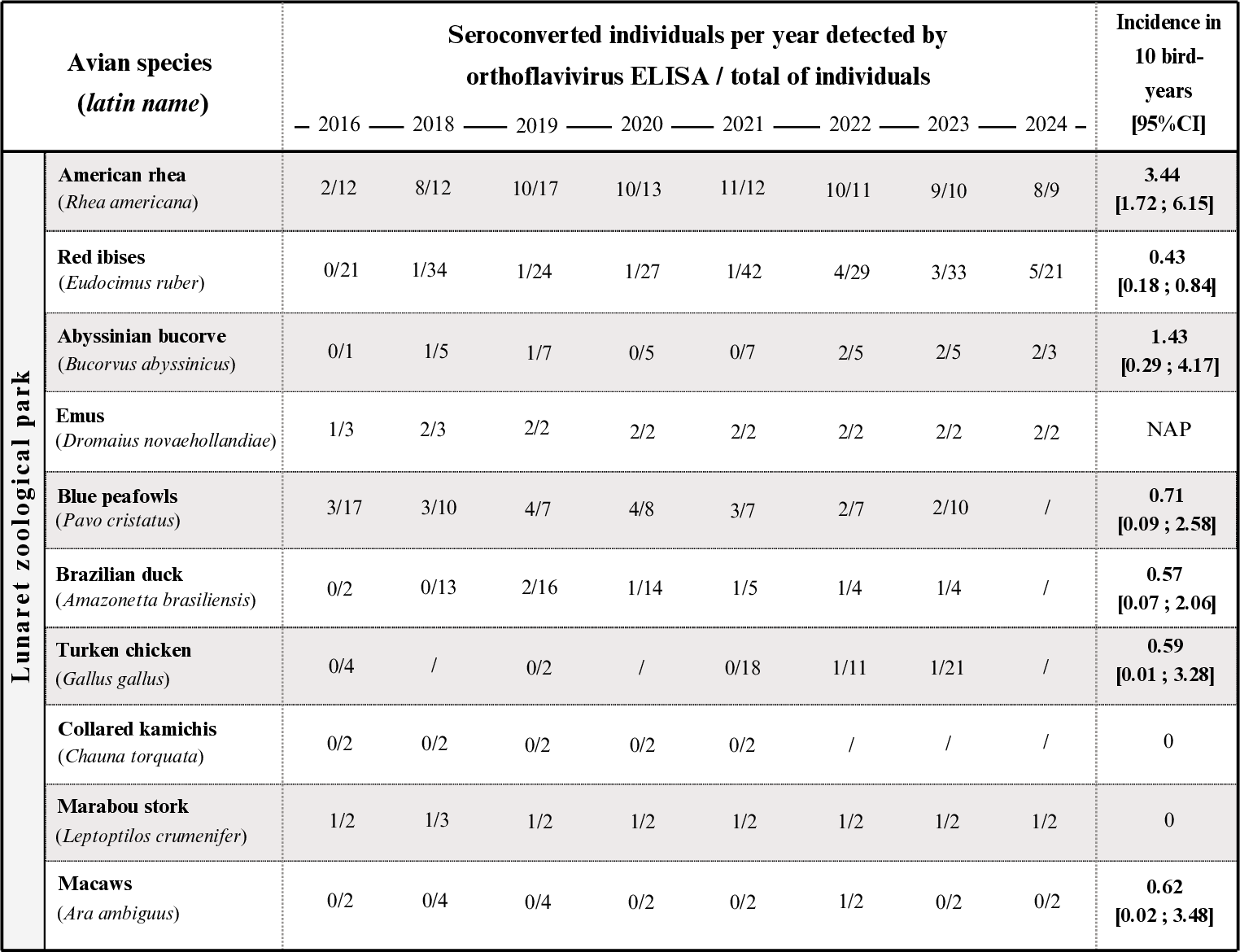

Supplement: S1 Table — (TIF) [file pntd.0013506.s001.tif]

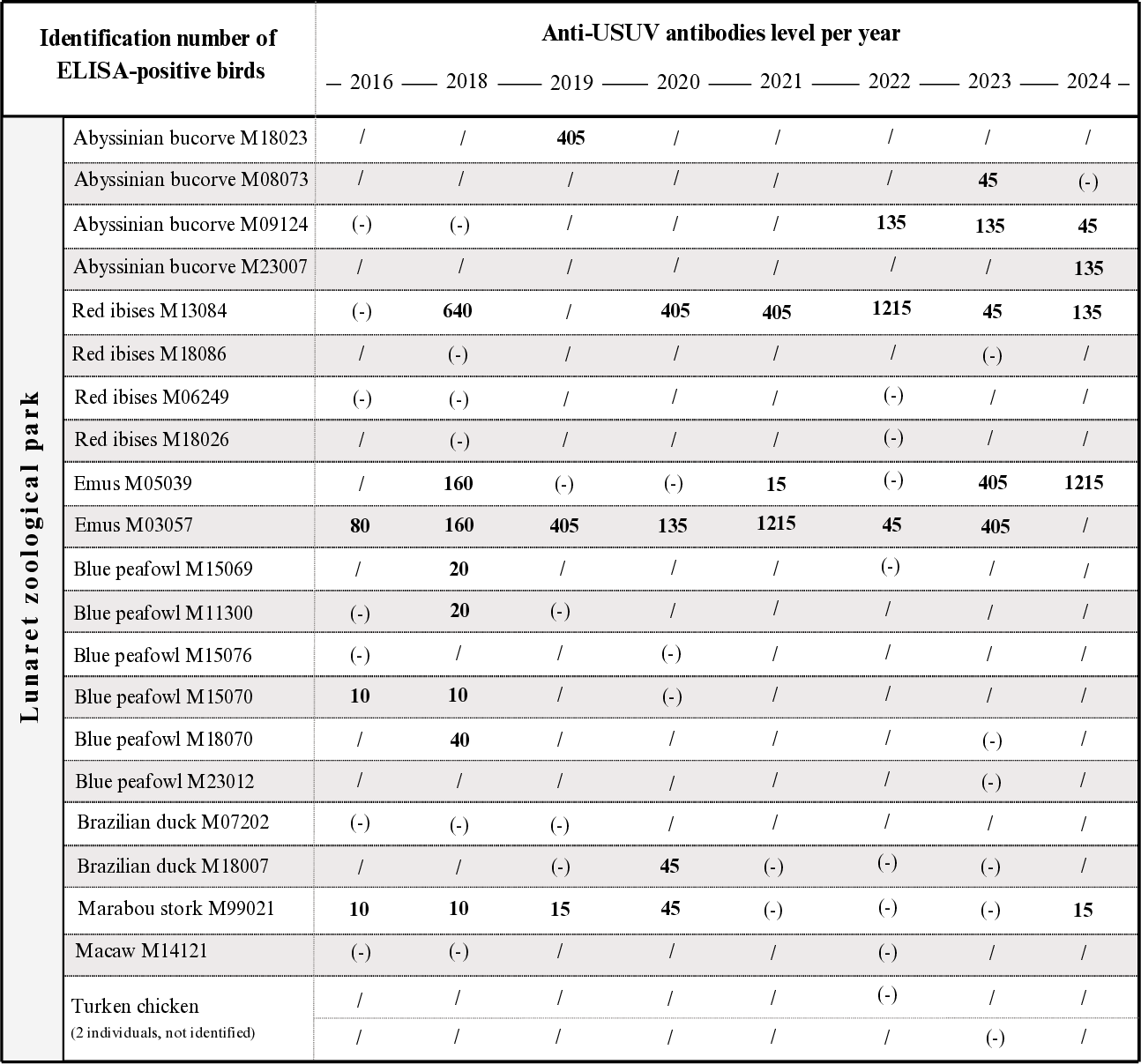

Supplement: S2 Table — (+) = presence of anti-USUV antibodies in sera; (-) = sample tested but absence of anti-USUV antibodies in serum;/ = sample not tested. (TIF) [file pntd.0013506.s002.tif]

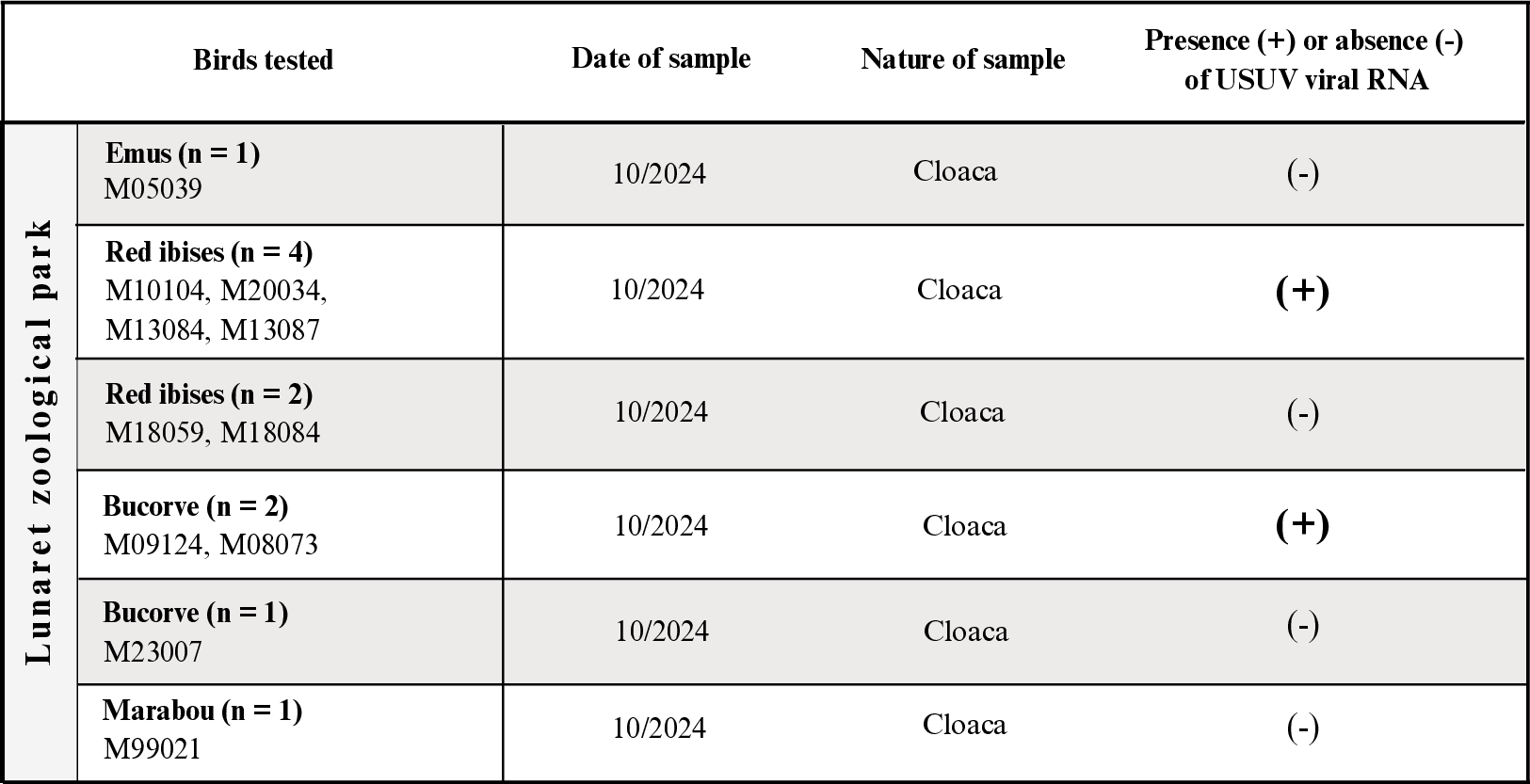

Supplement: S3 Table — (+) = Usutu viral RNA detected; (-) = no Usutu viral RNA detected. (TIF) [file pntd.0013506.s003.tif]
